# Non-variant specific antibody responses to the C-terminal region of merozoite surface protein-1 of *Plasmodium falciparum *(PfMSP-1_19_) in Iranians exposed to unstable malaria transmission

**DOI:** 10.1186/1475-2875-9-257

**Published:** 2010-09-16

**Authors:** Sedigheh Zakeri, Akram A Mehrizi, Samaneh Zoghi, Navid D Djadid

**Affiliations:** 1 Malaria and Vector Research Group (MVRG), Biotechnology Research Center, Institut Pasteur Iran, Pasteur Avenue, P.O.BOX 1316943551, Tehran, Iran

## Abstract

**Background:**

The C-terminal region of *Plasmodium falciparum *merozoite surface protein-1 (PfMSP-1_19_) is a leading malaria vaccine candidate antigen. However, the existence of different variants of this antigen can limit efficacy of the vaccine development based on this protein. Therefore, in this study, the main objective was to define the frequency of PfMSP-1_19 _haplotypes in malaria hypoendemic region of Iran and also to analyse cross-reactive and/or variant-specific antibody responses to four PfMSP-1_19 _variant forms.

**Methods:**

The PfMSP-1_19 _was genotyped in 50 infected subjects with *P. falciparum *collected during 2006-2008. Four GST-PfMSP-1_19 _variants (E/TSR/L, E/TSG/L, E/KNG/F and Q/KNG/L) were produced in *Escherichia coli *and naturally occurring IgG antibody to these proteins was evaluated in malaria patients' sera (n = 50) using ELISA. To determine the cross-reactivity of antibodies against each PfMSP-1_19 _variant in *P. falciparum-*infected human sera, an antibody depletion assay was performed in eleven corresponding patients' sera.

**Results:**

Sequence data of the PfMSP-1_19 _revealed five variant forms in which the haplotypes Q/KNG/L and Q/KNG/F were predominant types and the second most frequent haplotype was E/KNG/F. In addition, the prevalence of IgG antibodies to all four PfMSP-1_19 _variant forms was equal and high (84%) among the studied patients' sera. Immunodepletion results showed that in Iranian malaria patients, Q/KNG/L variant could induce not only cross-reactive antibody responses to other PfMSP-1_19 _variants, but also could induce some specific antibodies that are not able to recognize the E/TSG/L or E/TSR/L variant forms.

**Conclusion:**

The present findings demonstrated the presence of non-variant specific antibodies to PfMSP-1_19 _in Iranian falciparum malaria patients. This data suggests that polymorphism in PfMSP-1_19 _is less important and one variant of this antigen, particularly Q/KNG/L, may be sufficient to be included in PfMSP-1_19_-based vaccine.

## Background

*Plasmodium falciparum *is a major global health problem and is responsible for most cases of severe malaria and over one million deaths annually [1]. Increasing the drug-resistant *P. falciparum *strains [2,3] and also insecticide resistant Anopheles mosquito in different malaria-endemic regions of the world emphasizes the need for new controlling tools and strategies such as vaccine to combat *P. falciparum*.

Development of an effective vaccine against *P. falciparum *malaria has been a long-standing goal for malaria research and despite many decades of study, no effective vaccine against malaria parasite exists [4]. Genetic diversity in protective antigens is responsible for challenging in development of an effective malaria vaccine. This phenomenon will increase the parasite ability to evade immune responses, as a result, produce "vaccine-resistant parasite" and, therefore, threaten vaccine efficacy. To overcome the extensive genetic diversity in *P. falciparum *and develop protective vaccines, first, it is needed to understand the distribution of polymorphisms and then to measure allele-specific immune response to vaccine antigen in various endemic populations before conduction of vaccine trials.

Merozoite surface protein 1 (MSP-1) is the major protein on the surface of the blood stage of the parasite. Before erythrocyte invasion, the entire MSP-1 complex is shed, except for the C-terminal 19-kDa (MSP-1_19_), which remains on the surface as the merozoite enters the erythrocyte [5]. This fragment has been the focus of malaria vaccine development and consists of two epidermal growth factors (EGF)-like domains, each containing six cysteine residues [6], which are thought to have an important function in erythrocyte invasion [7,8]. *In vitro *and *in vivo *studies have shown that antibodies against PfMSP-1_19 _can prevent invasion of merozoites into red blood cells. These antibodies could block the cell cycle of parasites [9-14]. In addition, field studies also showed that naturally acquired antibodies to this antigen can inhibit erythrocyte invasion and, therefore, protect from clinical malaria [15-19].

Single nucleotide polymorphisms (SNPs) in PfMSP-1_19 _are caused limited sequence variations [20-22]. These mutations are at position 1644 (E/Q) in the first EGF domain and at positions 1691 (T/K), 1700 (S/N), 1701 (R/G) and 1716 (L/F) of the second EGF domain which lead to create different PfMSP-1_19 _variants (Q/KNG/L, E/KNG/L, E/KNG/F, Q/KNG/F, E/TSR/L, Q/TSR/L, Q/TSR/F, E/TSR/F, E/TSG/L etc.) that have been reported from global malaria endemic regions. Different studies have demonstrated cross-reactive antibody responses between PfMSP-1_19 _variant forms [16,23] with some specific recognition [16,23-25]. These specific antibody responses could be associated to polymorphic amino acids within the second EGF-like domain [16,23].

A study by Singh *et al. *[26] showed that immunized *Aotus *monkeys with PfMSP-1_19_-Q/KNG and/or PfMSP-1_19_- E/TSR variant(s) of PfMSP-1_19 _could develop antibodies to protect against challenge with *P. falciparum *Q/KNG parasite. Interestingly, limited studies have investigated the natural acquired immunity against different PfMSP-1_19 _variants in Kenya [23], India [27] and Peru [28] and have shown cross-reactivity in naturally acquired immunity arisen to PfMSP-1_19 _variants in spite of the presence of specific antibody responses.

Since diversity is encountered with development of effective malaria vaccine, having information on PfMSP-1_19 _genotype from clinical *P. falciparum *isolates and study on specific immunity to these variant forms in malaria endemic areas with different levels of transmission are important for selection which alleles to be included in multivalent vaccines. The earlier study conducted in malaria hypoendemic areas of Iran during 2001-2005, detected five PfMSP-1_19 _variants (E/TSR/L, E/TSG/L, E/KNG/F, Q/KNG/F and Q/KNG/L) with E/TSG/L as predominant form [22]. The main objective of this study was to define the frequency of PfMSP-1_19 _haplotypes over 2006 to 2008 in malaria hypoendemic regions of Iran and then to investigate cross-reactive and/or variant specific antibody responses to the circulating PfMSP-1_19 _forms. This study will help us to understand the natural antibody responses (variant specific or cross-reactive antibody responses) to different PfMSP-1_19 _variants of *P. falciparum *parasites that need to take into account in developing a polyvalent MSP-1_19_-based vaccine.

## Methods

### Subjects and blood sample collection

This study was performed in Sistan and Baluchistan province, Iran, where 10% of malaria patients suffered from falciparum malaria. The burden of malaria declined gradually over the last few years from 15,712 total cases in 2007 to 6,122 in 2009. In this region, most of the patients are adults and may experience several infections by *P. falciparum *and/or *Plasmodium vivax *with clinical symptoms. There is no record of severe malaria or death due to malaria. In this investigation, blood samples were collected from 50 *P. falciparum *infected individuals with symptomatic uncomplicated malaria attending at the Malaria Health Center in Chabahar Public Health Department in Sistan and Baluchistan province, south-eastern Iran during 2006 to 2008, as determined by Giemsa-stained thick smears. Age of the patients ranged between 5 to 75 years (mean 26.96) and 42 and 8 were male and female, respectively. Two ml venous blood was collected in tubes containing EDTA, and after centrifugation at 4,000 rpm for 10 min, plasma was collected and stored at -20°C until use. The study was explained in detail to all participants, and either they or their parents or legal guardians gave their signed informed consent. This study was approved by the Ethical Review Committee of Research in Institut Pasteur Iran.

### PCR amplification and sequencing of PfMSP-1_19_

Parasite genomic DNA was prepared by using the commercially available DNA purification kit (Promega, Madison, WI, USA) and kept at -20°C until use. Detection of *Plasmodium *species in samples was performed by both microscopy and nested-PCR amplification as described previously [29]. To define the sequence polymorphism in *Pfmsp*-*1*_*19 *_gene (corresponding to 4894 to 5242 bp) the following primers were used:

PfF: TCCAAggATCCTTAAACATTTCACAACAC

PfR: TCCTACTCgAgTTAAATgAAACTgTATAAT

Amplified fragments (n = 50) were gel-purified using the QIAGEN DNA purification kit (Qiagen, Germany) following the manufacturer's instructions. Direct sequencing of the DNA fragments was performed in both directions for each PCR product using the dideoxy chain termination procedure (Chemistry V3.1, Applied Biosystems) and also the 3730XL DNA analyser (Applied Biosystems) by MilleGen sequencing service (Labege, France). Nucleotide and amino acid sequences were aligned and compared by using ClustalX (with manual editing), with the published sequences, K1 (X03371) and MAD20 (X05624). The representing nucleotide sequences data of different *Pfmsp*-*1*_*19 *_variants reported in this article, were submitted to EMBL, GenBank and DDJB databases under the accession numbers HM569746-HM569750.

### Recombinant PfMSP-1_19_

The PfMSP-1_19 _variants (E/TSR/L, E/TSG/L, E/KNG/F and Q/KNG/L) with GenBank accession nos. HM569746, HM569747, HM569748 and HM569750, respectively) were cloned and expressed in *Escherichia coli *as recombinant proteins fused to the C-terminus of glutathione S-transferase (GST) of *Schistosoma japonicum *using the pGEX-KG vector.

Briefly, overnight culture from a single colony of PfMSP-1_19 _specific *E. coli *was expanded in TB (pH 7.2) containing ampicillin (100 μg/ml) at 30°C with shaking (150 rpm) until optical density (OD) 0.6-0.7 at 600 nm was reached. The expression of GST-PfMSP-1_19 _was induced with 0.5 mM IPTG (Sigma, USA). The culture was further grown for 4 h, and the cell pellet was lysed on ice by sonication (Ultraschallprozessor, Germany) with 10 sonication cycles, each consisting of 20 s pulses at 20 s intervals. The bacterial lysate was centrifuged at 12,000 rpm at 4°C for 30 min and the supernatant was incubated with glutathione sepharose 4B resin (Amersham Biosciences, USA) at 4°C for one hour and the resin was packed into a column. The column was washed with a 10-column volume of PBS1X, pH 7.4. The bound protein was eluted with a buffer containing 50 mM Tris-HCl, 10 mM reduced glutathione, pH 8. The fractions containing PfMSP-1_19 _were desalted with Econo-Pac 10 DG columns (BioRad, USA) according to the manufacture's manual and then concentrated with a concentrator (Eppendorf, Germany). The elutes were analysed by SDS-PAGE on 12% gels under reducing conditions and the concentration of the protein was determined using Bradford assay by a spectrophotometer (Eppendorf, Germany). To confirm the recombinant protein, Western blotting was carried out by standard protocols, with *P. falciparum *infected human sera. Control GST protein was prepared from *E. coli *with only GST gene constructs.

### ELISA

Anti-PfMSP-1_19 _in patients' sera was evaluated by ELISA. In brief, Maxisorp flat-bottomed 96-well microplates (Grainer, Labortechnic, Germany) were coated with 100 ng/well of any of the four recombinant affinity-purified GST-PfMSP-1_19 _(E/TSR/L, E/TSG/L, E/KNG/F, and Q/KNG/L) or with GST alone in 0.06 M carbonate-bicarbonate buffer (pH 9.6) and incubated at 4°C overnight. The plates were washed with PBS-Tween and blocked with 2% bovine serum albumin (BSA) for one hour at room temperature (RT). A volume of 100 μl of diluted plasma of individual patient plasma (1:200) was added in duplicate to coated plates and incubated for 2 h at RT. After washing, the plates were incubated with 100 μl of horseradish peroxidase-conjugated goat anti-human IgG Abs (1:25,000, Sigma, USA). The enzyme reaction was developed with o-phenylediamine dihydrochloride-H_2_O_2 _(Sigma, USA) and stopped with 2N H_2_SO_4_. The OD was measured using a microplate reader (Biotech, USA) at 492 nm, and the mean value of each pair of wells (after substraction of OD of GST) was calculated. A pool of adult Iranian immune sera from malaria endemic region with patent *P. falciparum *infection and 35 serum samples from healthy non-exposed Iranian from outside malaria endemic regions were used as positive and negative controls, respectively, in each plate. The cut-off value for positive plasma was considered as the mean plus 3 standard deviation (SD) of OD readings of negative samples (n = 35).

### Immunodepletion ELISA

To determine the cross-reactivity of antibodies against each PfMSP-1_19 _variants in *P. falciparum *infected human sera, an antibody depletion assay was performed in eleven corresponding patients' sera as described earlier [27]. Briefly, the 96-well primary plates were coated with 100 ng/well of each of PfMSP-1_19 _recombinant antigen, namely E/TSR/L, E/TSG/L, E/KNG/F and Q/KNG/L antigens and blocked with 3% BSA in PBS1X for one hour at RT. After blocking, all the plates were washed with 0.05% Tween 20 in PBS and then with PBS (pH 7.4). The first two wells in the first column were incubated for 30 min with sera of the respective sequence variants at a dilution of 1:1000 in PBS-T containing 1% BSA, while the remaining wells contained only wash buffer. After 30 min, the sera from the first two wells were transferred into the next wells in the second column and incubated for another 30 min. These serial incubations were carried out until all of the antibodies with a particular variant antigen were depleted, as determined by a standard ELISA. In this study, it was observed that antigen specific antibodies were completely removed from the respective sera after ten serial transfers. After the last transfer, the antibody-depleted sera were transferred into the secondary ELISA plates coated with the four PfMSP1_19 _variants (100 ng of each variant/well) and incubated for 30 min. Finally, the wells of both the primary and secondary plates were washed three times with PBS-T and incubated with horseradish peroxidase-labeled anti-IgG immunoglobulin (Sigma, USA) for one hour and the ELISA was completed as described above. The reactivity of each depleted sera with the other three haplotypes was compared with that of the undepleted sera.

### Statistical analysis

A database was created with SPSS 16.0 for windows (SPSS Inc., USA). The Spearman's correlation test was used to correlate antibody levels in all four PfMSP-1_19 _variant types. Kraskall-Wallis test was also performed to compare the antibody levels with corresponding infected-variant forms. The non-parametric Friedman test was applied to compare the variances between the levels of IgG responses to all four variants. In addition, Wilcoxan test was used to compare the IgG levels between two variant types for paired data. In all tests, *P *value < 0.05 was considered significant.

## Results

### Genotyping of *Pfmsp-1*_*19 *_gene

In this study, sequence comparison of the 19-kDa fragment of MSP-1 in a natural population of *P. falciparum *(n = 50) in Iran with the corresponding sequences of the PfMAD20 (X05624) and PfK1 (X03371) showed five distinct haplotypes, namely E/TSR/L (n = 10), E/TSG/L (n = 3), E/KNG/F (n = 11), Q/KNG/F (n = 13) and Q/KNG/L (n = 13). The *Pfmsp-1*_*19*_-Q/KNG/F and *Pfmsp-1*_*19*_-Q/KNG/L variant alleles were predominant haplotypes.

### Antibody responses to PfMSP-1_19 _variants

**Figure 1:**
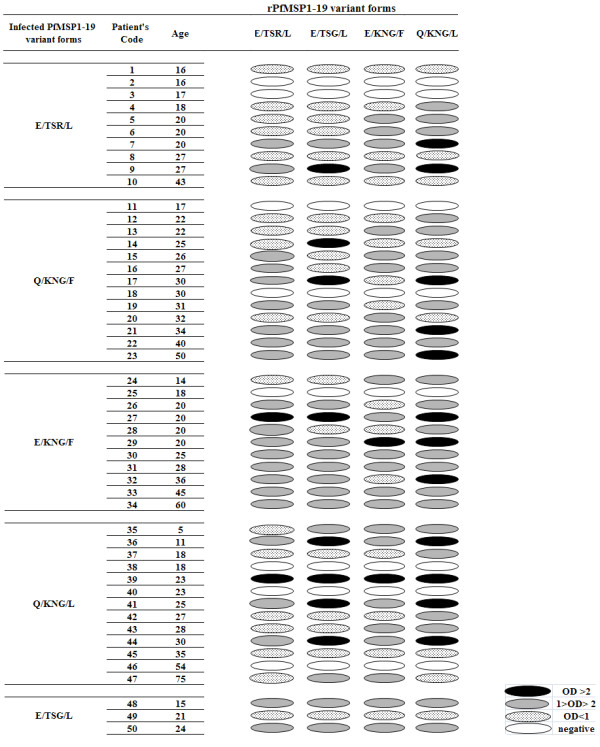
**Patterns of total IgG responses to four PfMSP-1**_**19 **_**variants in Iranian individuals infected with *P*. *falciparum *isolate**. Infected PfMSP-1_19 _variant forms in each patient were detected by sequencing analysis. Ages are given in years. Antibody reactivity of each sample is measured by ELISA. Cut-off values are 0.2, 0.3, 0.37 and 0.26 for IgG responses to E/TSG/L, E/TSR/L, E/KNG/F, and Q/KNG/L variants, respectively. The OD means values for IgG responses have been divided into the following groups: OD > 2: High-positive antibody responses. 1 > OD > 2: Medium-positive responses. OD < 1: Low-positive responses. OD < Cut-off: Negative.

All the four GST-PfMSP-1_19 _variants were expressed in *E. coli *in a soluble form and the purified proteins were analysed by SDS-PAGE and showed a molecular mass of ~37 kDa. Total IgG antibody responses to all four variants of PfMSP-1_19 _were analysed on 50 sera samples, indicating high, medium and low positives as well as negative values for each variant in a pair-wise manner (Figure 1). Of the 50 individuals, 42 (84%) and 8 (16%) were positive and negative for all allele variants, respectively. These eight negative responders harboured parasites with E/TSR/L (n = 2), Q/KNG/F (n = 2), E/KNG/F (n = 1) and Q/KNG/L (n = 3) haplotypes (Figure 1). Overall, no clear association was found between the five PfMSP-1_19 _variants of infecting *P. falciparum *parasite and antibody response among studied patients (*P *> 0.05, Kruskall-wallis; Figure 1).

The highest OD mean (± SD) value was for Q/KNG/L (1.54 ± 0.694, cut-off 0.26), followed by E/TSG/L (1.25 ± 0.663, cut-off 0.2) and E/KNG/F (1.22 ± 0.631, cut-off 0.37) and E/TSR/L (1.09 ± 0.566, cut-off 0.3). Comparing values of the IgG for all four PfMSP-1_19 _variants indicated a significant difference (*P *= 0.003, Friedman statistic = 14.13). However, the comparison between two variant forms showed a significant difference between E/TSR/L and Q/KNG/L (*P *< 0.0001), E/TSG/L and Q/KNG/L (*P *< 0.0001), E/KNG/F and Q/KNG/L (*P *= 0.044), using Wilcoxan test (Table 1).

**Table 1:** Frequency of positive antibody responses to four PfMSP-1_19 _variant forms in a pair-wise manner.

**PfMSP-1**_**19 **_**variants**	Pair-wise manner (%)	*P *value (Wilcoxan test)
E/TSR/L-E/TSG/L	39/50 (78)	= 0.273

E/TSR/L-E/KNG/F	35/50 (70)	= 0.251

**E/TSR/L-Q/KNG/L**	**30/50 (60)**	**< 0.0001**

E/TSG/L-E/KNG/F	31/50 (62)	= 0.735

**E/TSG/L-Q/KNG/L**	**31/50 (62)**	**< 0.0001**

**E/KNG/F-Q/KNG/L**	**31/50 (62)**	**= 0.044**

*P *value < 0.05 was considered significant.

The highest responses matched for E/TSR/L and E/TSG/L (78%) and E/TSR/L and E/KNG/F (70%) (Table 1); however, the E/KNG/F matched Q/KNG/L 62% of the time. In addition, in a paired correlation analysis comparing the continuous measurements to the four allele variants, E/TSR/L and E/TSG/L were found at the highest rate (*r *= 0.866) and the lowest correlation coefficient was found to be between E/TSG/L and E/KNG/F (*r *= 0.670). Correlations between all pairs were significant (*P *< 0.0001, Table 2).

**Table 2:** Correlation analysis of IgG antibody responses in four PfMSP-1_19 _variant forms

**PfMSP-1**_**19 **_**variants**	Correlation coefficient	*P *value
E/TSR/L-E/TSG/L	*r *= 0.866	< 0.0001

E/TSR/L-E/KNG/F	*r *= 0.738	< 0.0001

E/TSRL-Q/KNG/L	*r *= 0.844	< 0.0001

E/TSG/L-E/KNG/F	*r *= 0.670	< 0.0001

E/TSG/L-Q/KNG/L	*r *= 0.839	< 0.0001

E/KNG/F-Q/KNG/L	*r *= 0.709	< 0.0001

The *P *value denotes the Spearman correlation coefficient. *P *value < 0.05 was considered significant.

### Cross-reactive and variant-specific responses

**Figure 2:**
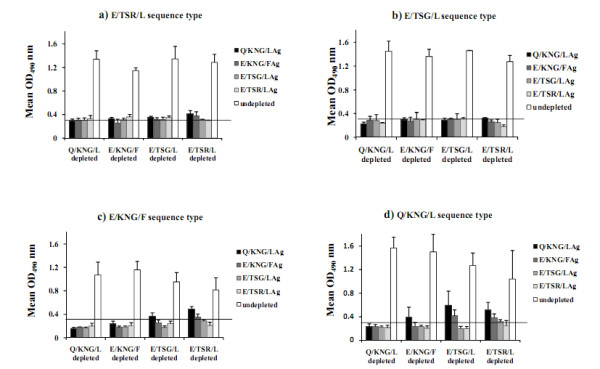
**Immunodepletion results showing cross-reactive and specific antibodies in the sera of infected patients with *P. falciparum *isolates**. a) Sera from patients infected with E/TSR/L sequence type (n = 3), b) E/TSG/L sequence type (n = 2), c) E/KNG/F sequence type (n = 3) and d) Q/KNG/L sequence type (n = 3). The cross-reactive antibodies between antigens were depleted with all four variant forms. The mean antibody OD values are shown and error bars indicate the SD. The horizontal line shows the cut-off values.

In this investigation, to determine the cross-reactive and variant-specific responses to the four PfMSP-1_19 _variants during natural infection, an ELISA depletion assay was used. In this assay, the cross-reactive antibodies between antigens were depleted with all the variants (74% to 85% reduction in reactivity) and therefore, only antibodies to different sites of antigen remain to bind in secondary plate. Sequencing analysis showed that the eleven examined patients harboured different PfMSP-1_19 _variants and also they had a high level of total IgG against four PfMSP-1_19 _variants (Figure 2). To indicate type-specific antibody responses to Q-/E-, -TSR/-TSG/-KNG, all the eleven examined patients' sera were depleted by four examined variants and the result showed that sera depleted with Q/KNG/L allele type did not have a positive antibody response to any of four allele types (Figure 2). However, sera depleted with either E/TSR/L or E/TSG/L allele types recognized Q/KNG/L and E/KNG/F, indicating specific antibody responses to Q- and/or -KNG (Figure 2). With respect to the Q- specific antibody responses, depleted sera with E/KNG/F recognized Q/KNG/L allele type. Overall, it was observed that after antibody depletion with all four variant forms, eight patients had low-level specific antibody to Q- and -KNG. Of these eight sera samples, 7 and 8 had specific antibody responses to -KNG and Q-, respectively.

## Discussion

The development of MSP-1 vaccine has focused on the C-terminal 19-kDa fragment (PfMSP-1_19_) and antibodies against this antigen may block parasite invasion into erythrocytes, leading to reduction in parasitaemia and thus protection against the disease [9,30]. This antigen is also recognized by human antibodies [31] that could reduce the risk of clinical malaria [15,16,19,32,33]. The PfMSP-1_19 _is a highly conserved antigen, but the presence of limited polymorphisms in EGF-like domains of this fragment might compromise its use as a vaccine candidate. Therefore, the purpose of this study was whether sequence polymorphisms in *Pfmsp-1*_*19 *_in *P. falciparum *isolates from malaria hypoendemic regions in Iran influence the recognition and specificity of naturally occurring antibody to the four sequence types of PfMSP-1_19 _antigens (E/TSR/L, E/TSG/L, E/KNG/F and Q/KNG/L), since protective immunity is thought to target this molecule.

Sequence data of the PfMSP-1_19 _revealed that the haplotypes Q/KNG/L and Q/KNG/F were predominant variant forms and the second most frequent haplotype was E/KNG/F during 2006- 2008. However, an earlier molecular study, in the same region revealed five sequence patterns, in which E/TSG/L variant form was the predominant haplotype among Iranian isolates during 2001-2005 [22]. Although, in this study, the Q/KNG/L-bearing parasites are predominant over E/TSR/L or E/TSG/L parasites, the prevalence of antibodies to all four PfMSP-1_19 _variant forms was equal and high (84%). The reason could be that Iranian malaria patients were often exposed to mixed parasite variant sequences of PfMSP-1_19 _over time and developed specific polyclonal antibodies against multiple variants in a mixed infection. Regarding to seronegative individuals, three of them had first exposure to the *Plasmodium *infection and the rest (n = 5) have not been infected with this parasite (based on questionnaire) recently and, in fact, in the absence of boosting, antibody responses reduce to a detectable level.

In malaria endemic regions, it is difficult to distinguish specific antibodies responses from antibodies to conserved determinants of antigen in naturally infected individuals who were exposed to mixed parasite variant sequence over time. This may be further complicated if the past histories of infection remain unclear. To evaluate the variant specific responses to PfMSP-1_19_, the IgG antibody to each of the examined variants was analysed using immunodepletion assay. The results showed that Q/KNG/L-infected sera depleted with the same variant were unable to diagnose other PfMSP-1_19 _variant forms. However, if it depleted with E/TSR/L, E/TSG/L or E/KNG/F variants, IgG antibody response was observed against Q/KNG/L that could be explained by specific IgG response to epitope containing Q- (at 1644) residue. It is also important to note that E/KNG/F-infected sera depleted with E/TSR/L variant were able to diagnose Q/KNG/L but not E/KNG/F variant because of the existence of antibody specific response to epitope containing Q- (at 1644) residue. This might be explained by past infection of these individuals with Q/KNG/L variant form. In addition, Q/KNG/L-infected sera depleted with E/TSG/L and E/TSR/L had low-positive IgG response to E/KNG/F due to the specific response to epitope containing -KNG (at 1691, 1700 and 1701) residues, which is highly exposed to immune system [34,35]. However, based on the previous studies, the -F/-L (at 1761) residue is located in the inter-domain region of the two EGF domains, with limited surface exposure [34,35] and therefore, dose not seem to contribute as a major B-cell epitope. In total, the results showed that in Iranian individuals who were infected with different PfMSP-1_19 _variants could induce not only cross-reactive antibody responses to heterologous variants but also could induce some specific antibodies to homologous variant. Moreover, E/TSG/L and E/TSR/L did not induce specific antibody responses to epitopes containing E- (at 1644), -TSG (at 1691, 1700 and 1701) or -TSR (at 1691, 1700 and 1701) residues. Therefore, it is suggested that the majority of antibody responses have sharing B-cell epitopes and the variant- specific immune response is to epitopes containing Q- (at 1644) and in somewhat to -KNG (at 1691, 1700 and 1701) residues. Although the earlier studies suggested that antibodies to PfMSP-1_19 _are associated with protection [16,17,26,36], further study is required to analyse whether these non-variant antibody responses to PfMSP-1_19 _are associated with clinical protection and parasitaemia.

## Conclusion

In summary, due to the presence of antigenic diversity in vaccine antigen, it may be necessary to develop a polyvalent vaccine that would increase the cost of developing and manufacturing vaccines. Therefore, it may be possible to select an antigen with limited polymorphisms that its variant forms are recognized by cross-protective antibodies. In this investigation, Iranian individuals who were exposed to *falciparum *malaria develop IgG antibodies to conserved B-cell epitopes of different PfMSP-1_19 _variant forms that this is in line with the previous studies in Kenya [17,23], India [27] and Peru [28]. The present findings also suggest that possibly, polymorphisms in PfMSP-1_19 _antigen could be less important and one variant of this antigen, especially widely distributed Q/KNG/L, may be sufficient to be included in PfMSP-1_19_-based vaccine. The impact of these cross-reactive antibodies to block parasites invasion into the erythrocyte needs further investigation to be elucidated.

## Competing interests

The authors declare that they have no competing interests.

## Authors' contributions

SZ* designed the work and supervised the study, set up the ELISA depletion protocol, analysed the data and wrote the manuscript. AAM carried out polymorphism studies, expressed the proteins and performed the ELISA depletion assay. SZ contributed to ELISA depletion assay. NDD participated in sample collection, helped with the sequence data analysis and also critical reading of the manuscript. All authors read and approved the final manuscript and agreed the submission.
